# Favipiravir Protects Enterocytes From Cell Death After Inflammatory Storm

**DOI:** 10.7759/cureus.47417

**Published:** 2023-10-21

**Authors:** Ugur Ozgurbuz, Hilal Kabadayi Ensarioglu, Damla Akogullari Celik, Hafize Seda Vatansever

**Affiliations:** 1 Anesthesiology and Reanimation, Izmir Ataturk Research and Training Hospital, İzmir, TUR; 2 Histology and Embryology, Manisa Celal Bayar University, Manisa, TUR

**Keywords:** necroptosis, apoptosis, enterocytes, inflammatory bowel disease, inflammation, favipiravir

## Abstract

Over the past years, inflammatory bowel disease (IBD) treatment has become more targeted, anticipating the use of immune-modifying therapies at an earlier stage. During the treatment process prevention and management of viral infections hold significant importance.

The protective role of favipiravir on enterocytes which are affected by inflammation is still unknown. We aim to analyze the effects of favipiravir on enterocytes after an inflammatory condition.

We conducted a 2,5-diphenyl-2H-tetrazolium bromide (MTT) assay to assess the cytotoxicity of favipiravir on intestinal epithelioid cells (IEC-6). To mimic the inflammation model in cell culture conditions, we exposed IEC-6 cells to tumor necrosis factor-α (TNF-α) and interferon-γ (IFN-γ). The cells were categorized into four groups: control, inflammation model, application of favipiravir before inflammation (prophylactic), and application of favipiravir after inflammation (treatment). We assessed the presence and distribution of caspase 1, caspase 3, interleukin 6 (IL6), interleukin 8 (IL8), mixed lineage kinase domain-like protein (MLKL), receptor-interacting protein kinase 1 (RIPK1), and TNF-α using indirect immunoperoxidase staining. TNF-α and IL8 levels were analyzed with enzyme-linked immunosorbent assay (ELISA) in a culture medium.

Caspase 1 was observed to be strong (+++) in the treatment group and weak (+) in the prophylactic group compared to the inflammation group. Caspase 3 was weak (+) in the inflammation group, and it was strong (+++) in the prophylactic and treatment group, the increase in the treatment group was significant. Therefore administering favipiravir before inducing inflammation appears to control the inflammatory caspase pathway in intestinal enterocytes, protecting them from inflammatory responses, while the caspase 3-dependent apoptotic pathway may not be active in enterocytes during inflammation. IL6 and IL8 were negative (-) in control, IL6 was weak (+) in inflammation and favipiravir treated groups; IL8 increased significantly in favipiravir groups compared to control and inflammation groups. Consequently, favipiravir may trigger IL6 release, initiating the inflammatory pathway and potentially enhancing IL8 interactions with other cytokines. TNF-α immunoreactivity was strong (+++) in the inflammation group, while it was moderate (++) in favipiravir-administered groups. MLKL immunoreactivity was strong (+++) in all groups, RIPK1 was weak (+) in control, strong (+++) in the inflammation and treatment group, moderate (++) in the prophylactic group, and the increase in inflammation and treatment group was significant compared to control. Our findings suggest that in the treatment group, necroptosis was triggered by increased MLKL and RIPK1, key players in inflammation and cell death.

After immunocytochemical evaluation, our findings suggest that, after the onset of inflammation, favipiravir may play a role in cell death by increasing necroptosis rather than apoptosis.

## Introduction

Inflammatory bowel disease (IBD) refers to a group of chronic inflammatory disorders that target the gastrointestinal (GI) tract. The primary classifications of IBD encompass ulcerative colitis (UC) and Crohn's disease (CD). UC is often characterized by surface-level inflammation in the rectum that can extend into nearby mucosal regions in a continuous pattern. Conversely, CD involves inflammation penetrating through the layers of the GI tract and can manifest in any segment of it [1].

Globally, recent research indicates a rising occurrence of IBD [2]. In 2018, within North America, an annual report recorded up to 11.4 new instances per 100,000 individuals, particularly affecting children and adolescents under 18, who constitute 25% of all IBD cases [2].

Several studies have described that the intestinal microbiota and microbial dysbiosis might contribute to the development of IBD [3].

Interactions between viral infections and IBD can vary based on factors such as the specific virus involved, the severity of the underlying IBD, and the overall health status of the patient [4-6]. While bacterial elements are more commonly linked with IBD, viral infections like rotavirus, norovirus, and herpesviruses like cytomegalovirus and severe acute respiratory syndrome coronavirus 2 (SARS-CoV-2) are identified as culprits behind intestinal pathologies and associated symptoms, including inflammation, abdominal pain, and diarrhea [4-6]. Also, Sencio et al. showed that viral respiratory infection which is caused by influenza, can remotely trigger intestinal disorders (namely, mild inflammation and altered barrier functions) [7].

Furthermore, individuals within the IBD population who undergo immune-modifying treatments face an elevated susceptibility to severe infections, particularly those stemming from viral agents [8]. Among these opportunistic infections, 40% can be attributed to viral pathogens, encompassing hepatitis A virus, hepatitis C virus, hepatitis B virus, human papillomavirus, influenza virus, human immunodeficiency virus, herpes simplex virus, cytomegalovirus, varicella-zoster virus, Epstein-Barr virus, and the SARS-CoV-2 (which causes coronavirus disease 19) [8]. Notably, viral infections represent demanding problems for the clinician, as they are often difficult to diagnose and are associated with significant morbidity [8].

Favipiravir is a type of ribonucleic acid (RNA)-dependent RNA polymerase inhibitor that blocks RNA virus replication [9-11]. It works by inhibiting the replication of the virus within the host cells [12].

Upon entry into the cell, favipiravir activates to form ribofuranosyl-5ʹ-triphosphate (favipiravir-RTP) by entering intracellular phosphoribosylation and phosphorylation, displacing purine nucleosides and creating a substrate for the RNA-dependent RNA polymerase (RdRp) enzyme [13]. In this way, it inhibits the RdRp enzyme and prevents the elongation of the viral RNA chain and protein synthesis [13]. Following virus replication in the cell, the production of type 1 interferon (IFN) increases, such as interleukin (IL)-1β, IFN-γ, tumor necrosis factor-alpha (TNF-α), and monocyte chemoattractant protein-1 (MCP-1) [13]. Since RdRp domains are not present in human cells and are conserved among RNA viruses, this distinct specific mechanism targeting RNA viral polymerases makes favipiravir an attractive drug candidate.

Although favipiravir is not typically recognized as having a direct role in inflammation, it is important to acknowledge that inflammation can be a response to viral infections [14,15]. Following infection, immune system cells such as macrophages and neutrophils are activated, leading to the secretion of pro-inflammatory cytokines and chemokines that in turn activate T helper cells [14,15]. This cascade of events triggers an escalation in inflammation, which can result in cellular damage within tissues and organs [14,15]. However, the secretion of certain anti-inflammatory cytokines like interleukin 4 (IL4) and interleukin 10 (IL10) works to counteract the inflammatory process [14,15]. Besides this, interleukin 6 (IL-6) aids in the body's defense against infections and injuries, orchestrating acute phase responses and immune reactions. However, unregulated IL-6 synthesis can lead to chronic inflammation and autoimmunity. Moreover, interleukin 8 (IL-8) acts as a proinflammatory cytokine, promoting angiogenesis and cell motility, and is involved in the acute inflammatory response, activating various cells including neutrophils [14,15].

Interestingly, a number of viruses express molecules that are capable of directly interfering with the host cell death machinery, suggesting that these viruses might have the potential to disrupt the balance between cell death and proliferation in the gut [5]. To illustrate, SARS-CoV-2 induces significant apoptosis in diverse host cells, resulting in extensive tissue damage and functional loss, thereby hastening disease progression and potentially leading to mortality [6].

A cytokine storm is an intense immune response characterized by the rapid and excessive release of cytokines into the bloodstream [16]. While cytokines are crucial for normal immune functions, their overproduction can have detrimental effects [16]. In response to this situation, organ damage, including intestinal injury, can occur [16]. Furthermore, the excessive release of cytokines may activate inflammatory cell death pathways such as pyroptosis and necroptosis. Caspase 1 primarily regulates the inflammatory response, while recent research indicates its role in pyroptosis. Caspase 3, on the other hand, is crucial in executing apoptosis, breaking down cellular components in a controlled manner. Both caspase 1 and caspase 3 contribute to the regulated process of cell death, ensuring the removal of unwanted cells without causing excessive damage to surrounding tissue [6].

Necroptosis is caspase-independent cell death and the formation of necrosome was assembled by death ligands such as TNF-α, Fas ligand (FasL), IFN-γ, caspase 8, receptor-interacting protein kinase 1 (RIPK1) and receptor-interacting protein kinase 3 (RIPK3) [17]. After autophosphorylation of RIPK1 and RIPK3, phosphorylate mixed lineage kinase domain-like protein (MLKL) leads to cell death and release of cytokine. Yet, managing virus-triggered cell death stands as an alternate strategy to safeguard against tissue damage.

In recent years, there has been a shift towards more targeted treatment for IBD, with a growing emphasis on the early use of immune-modifying therapies. In the course of treatment, the prevention and management of viral infections have gained considerable importance. However, the specific protective effects of favipiravir on enterocytes affected by inflammation remain unclear. Our objective is to examine the impact of favipiravir on enterocytes under inflammatory conditions.

## Materials and methods

In our study, an in vitro inflammation model was created by applying TNF-α and IFN-γ to intestinal epithelioid cells (IEC-6). The dose of favipiravir was determined by evaluating its cytotoxicity in IEC-6 cells with 2,5-diphenyl-2H-tetrazolium bromide (MTT). IEC-6 cells were divided into four groups: control, inflammation model, prophylactic group administered favipiravir before inflammation, and treatment group administered favipiravir after inflammation. The presence and distribution of caspase 1, caspase 3, IL6, IL8, MLKL, RIPK1, and TNF-α were evaluated by indirect immuno-peroxidase staining method. Simultaneously, TNF-α and IL8 levels in culture media were analyzed by enzyme-linked immunosorbent assay (ELISA).

Ethical approval

In this study, we conducted in vitro cell line research, which does not involve human subjects or animals. As such, ethical approval was not required for this study due to its non-human, laboratory-based nature. The research was conducted in compliance with all relevant institutional and national guidelines.

Cell culture

IEC-6 cells (intestinal epithelioid cell line, CRL-1592, ATCC) were used in this study. They were cultured with 90% fetal bovine serum (FBS, 10270-106, Gibco), 1% penicillin-streptomycin (PS-B, Capricorn Scientific) included 10% α-MEM (MEMA-RXA, Capricorn Scientific) in a humidified atmosphere at 37˚C and 5% CO_2_.

Cell viability assay: determination of the cytotoxic dose of favipiravir on enterocytes

The IEC-6 cells were seeded in 96 well culture dishes at a density of 2x10^3^ cells/mL and incubated for 24 hours. The following day, favipiravir (Favimol, Neutec) was diluted in accordance with human dosage (600 mg/L, 1200 mg/L, 1600 mg/L, and 2000 mg/L) in completed cell culture media and applied to the cells for 24h or 48h [6]. Cell viability was estimated by MTT assay. MTT solution (GC4568, Glentham Life Sciences) was prepared in 5 mg/mL of phosphate-buffered saline (PBS, PBS404.100, Bioshop). After the incubation period of the cells with favipiravir, 1/10 dilution of MTT was added to each well and incubated for 4 h at 37˚C. The process was stopped by adding 50 μl dimethyl sulphoxide (DMSO) (GK2245, Glentham Life Sciences) to each well. Absorbance was measured with a microplate reader (ELX800UV, BioTek Instruments Inc), using 540 nm as the reference wavelength.

Experimental groups and cell stimulation

IEC-6 cells were plated in 8-well plates at a density of 1x10^3^ cells per well and incubated for 48 hours. To induce an in vitro inflammation model mimicking a cytokine storm, we employed a combination of TNF-α and IFN-γ, following the protocols from prior studies [18-20]. The cells were categorized into four distinct groups for experimentation. The first group served as the control, where cells were cultured using the IEC-6 culture medium alone. The second group represented the inflammation model, in which TNF-α (50 ng/ml, 315-01A-50UG, PeproTech) and IFN-γ (100 ng/ml, 315-05-100UG, PeproTech) were administered for a 48-hour period, following the methodology outlined by Karki et al., 2021 [20]. For the third and fourth groups, the administration of favipiravir was carried out either before (prophylactic) or after (treatment) the application of TNF-α and IFN-γ, respectively.

Immunocytochemistry assay

Distributions of caspase 1, caspase 3, IL6, IL8, MLKL, RIPK1, and TNF-α were analyzed using indirect immunoperoxidase staining. The cells were fixed with 4% paraformaldehyde (1.04004.0800, Merck) for 30 min and were washed three times with PBS. They were then incubated for 15 min with 0.1% Triton-X-100 (A4975,0100, Applichem) solution on ice for permeabilization. Later, 3% hydrogen peroxide (H_2_O_2_, 1.08597.2500, Merck) was applied for 5 min after washing with PBS. The cells from all groups were then treated with blocking solution (TA-125-UB, ThermoFisher) for an hour at room temperature and were incubated with anti-caspase 1 (BT-AP01191, BT-LAB), anti-caspase 3 (BT-AP01199, BT-LAB), IL6 (SC-1265, SantaCruz), IL8 (BT-AP04514, BT-LAB), MLKL (BS-5513R, Bioss), RIPK1 (BS-5805R, Bioss) and TNF-α (BT-AP09103, BT-LAB) overnight at 4˚C. The cells were washed with PBS and incubated with a biotinylated rabbit anti-mouse secondary antibody (TP-125-UB, ThermoFisher) for 30 min. After washing with PBS, streptavidin-hydrogen peroxidase (TP-125-UB, ThermoFisher) was added for 30 min. To develop the immunocytochemical reaction, diaminobenzidine (DAB, 38611, ScyTek Laboratories) was applied for 5 min. After washing with PBS, Mayer’s hematoxylin was used as a counterstain and they were mounted with a mounting medium (DMM-125, Spring Bioscience). The intensity of immunolabeling was evaluated by the two investigators at different times with light microscopy (BX40, Olympus). The immunoreactivities were considered negative (-), weak (+), moderate (++), and strong (+++).

Enzyme-linked immunosorbent assay (ELISA)

ELISA to determine IL8 (SRB-T-83151, SunRedBio) and TNF-α (201-12-0083, SunRedBio) levels in all group's culture media was performed according to the kit manufacturer’s instructions. First, standards and culture media were prepared and added to coated 96 well plates. After that, biotin-containing IL8 or TNF-α antibodies, and streptavidin-HRP were added, respectively, and incubated at 37˚C for 60 min. Until this stage, no action was applied to the blank wells. After washing five times at the end of the incubation, substrates were put into wells and incubated in a lightless environment. Then stop solution was added into all wells to terminate the reaction at the end of the incubation, the measurement was performed at 450 nm absorbance with a microplate reader (ELX800UV, Biotek Instruments Inc). Levels were calculated according to the kit manufacturer’s instructions.

Statistical analysis 

All experiments were repeated three times. Data graphs were created via Graphpad Prism 8.0.1. Statistical evaluation was determined using a one-way analysis of variance (ANOVA) test. All data are expressed as mean ± SD, and *p* values *p*<0.05 were considered statistically significant.

## Results

Cell culture

**Figure 1:**
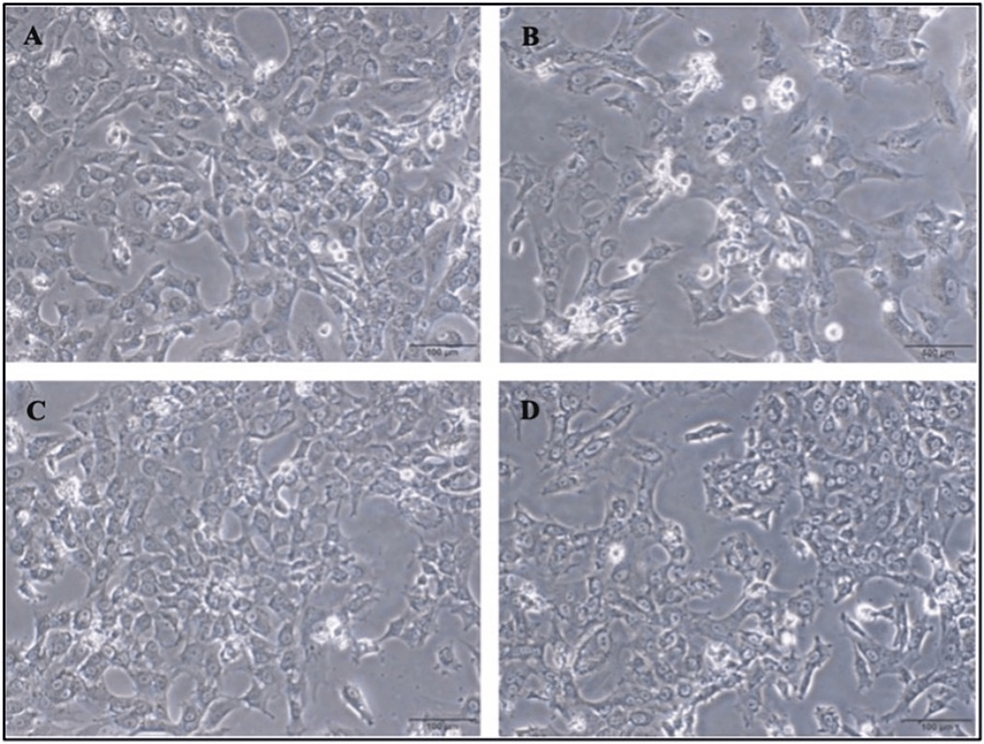
Cell culture of IEC-6 cells. Cell culture photographs of IEC-6 cells at control (A), inflammation model (B), prophylactic (C), and treatment (D) groups. Scale bars: 100 µm.

The epitheloid cell structure of IEC-6 cells was preserved in all groups (Figure 1), and the cytoplasmic deposits were present in both the inflammation model (Figure 1B) and treatment (Figure 1D) groups. In addition, the cell density in the prophylactic (Figure 1C) or treatment (Figure 1D) groups was similar to the control group (Figure 1A). 

Cell viability and cytotoxicity

**Figure 2:**
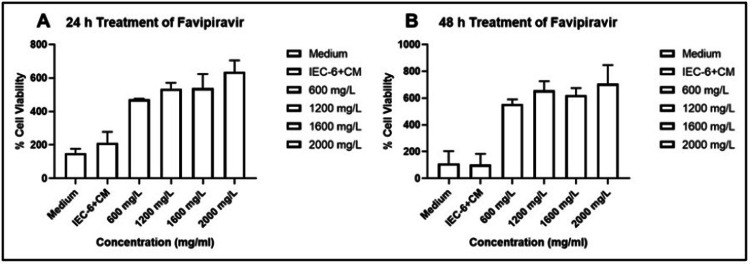
Cell viability assay of favipiravir in IEC-6 cells. Dose-response columns of favipiravir. Cell viability was quantitated by the MTT assay. IEC-6 cells were exposed to different concentrations of favipiravir for 24 (A) and 48 (B) hours. The results are presented as mean ± SD. After 24 h of favipiravir application to the IEC-6 cell lines; 600 mg/L: p>0.9999, 1200 mg/L: p>0.9999, 1600 mg/L: p>0.9999, 2000 mg/L: p=0.1324 compared to the IEC-6+CM group. After 48 h of favipiravir application to the IEC-6 cell lines 600 mg/L: p>0.9999, 1200 mg/L: p=0.4364, 1600 mg/L: p>0.9999, 2000 mg/L: p=0.2457 compared to the IEC-6+CM group. CM: Culture medium, MTT: 2,5-diphenyl-2H-tetrazolium bromide.

IEC-6 cells were treated with favipiravir at various dilutions (600 mg/L, 1200 mg/L, 1600 mg/L, and 2000 mg/L) for 24 h and 48 h. Cell viability was determined as described above by the MTT assay. All tested concentrations of favipiravir showed increased IEC-6 cell proliferation. Because of doubled number of cells were detected in 1600 mg/L dilution of favipiravir for both 24 h and 48 h treatment (Figure 2), in the rest of the study, this dilution was used for 24 h.

Levels of IL8 and TNF-α in culture medium from all groups 

**Figure 3:**
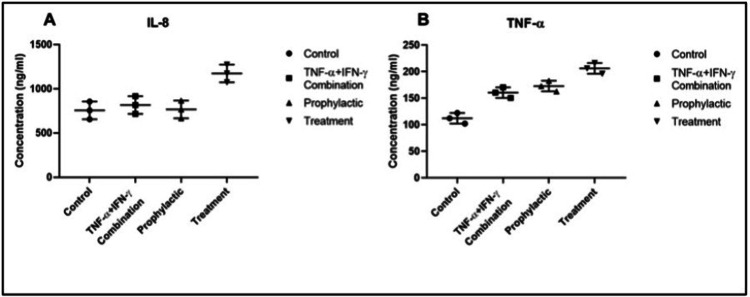
Evaluation of IL8 (A) and TNF-α (B) in study groups. Levels of IL8 (A) and TNF-α (B) in culture mediums from control, inflammation model, before (prophylactic) or after favipiravir applied (treatment) inflammation model groups after ELISA. The results are presented as mean ± SD. IL-8 and TNF-α levels were highest in the treatment group (*p*=0.0266 and *p*=0.0266 respectively). ELISA: Enzyme-linked immunosorbent assay.

IL8 and TNF-α levels in culture medium from all groups were evaluated by ELISA assay. The mean optical density (OD) value of IL8 for the control group was 755 ± 0.02, while the mean OD values for the inflammation model, prophylactic, and treatment groups were 817.22 ± 0.03, 767.22 ± 0.17, and 1172.77 ± 0.06 respectively. The mean OD value of TNF-α for the control group was 112.23 ± 0.01, while the mean OD values for the inflammation model, prophylactic, and treatment groups were 160.57 ± 0.07, 172.71 ± 0.17, and 206.04 ± 0.03 respectively. Both TNF-α and IL8 levels were highest in the treatment group enterocyte culture medium (TNF-α *p*=0.0266 and IL8, *p*=0.0266) (Figure 3). The IL8 level was lesser both in the inflammation model group (*p*=0.3467) and prophylactic group (*p*=0.3467) culture medium (Figure 3A). The level of TNF-α in the inflammation model group (*p*>0.9999) was less than the prophylactic group (*p*=0.3467) (Figure 3B).

Immunocytochemical evaluation

**Figure 4:**
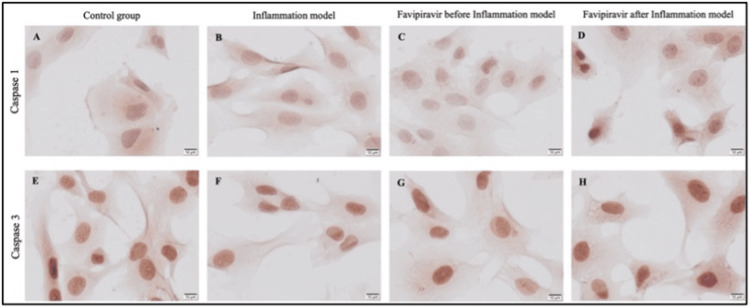
Evaluation of caspase 1 and caspase 3 through immunocytochemistry in the study groups. Immunoreactivity of caspase 1 (A-D) and caspase 3 (E-H) in control (A, E), inflammation model (B, F), before (prophylactic) (C, G) or after favipiravir applied (treatment) (D, H) inflammation model groups. Scale bars: 10 µm.

**Figure 5:**
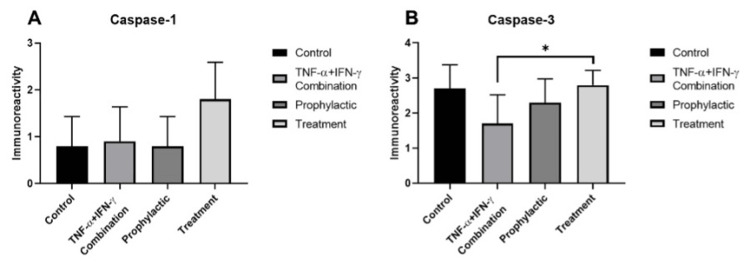
Statistical evaluation of the immunocytochemistry results for caspase 1 and caspase 3 across the study groups. Caspase 1 (A) and caspase 3 (B) immunoreactivity measurements and statistical analysis of them in all experimental groups. The results are presented as mean ± SD and significance of difference indicated with asterisk (*) as *p* value smaller than 0.05 (*p*<0.05).

Caspase 1 immunoreactivity was strongly positive (+++) in the treatment group (Figure 4D, Figure 5A) while it was moderate (++) in the control and inflammation model groups (Figure 4A, 4B, and Figure 5A). However weak (+) caspase 1 immunoreactivity was detected in the prophylactic group (Figure 4C, Figure 5A). After the immunocytochemistry assay, the caspase 3 immunoreactivity was moderate (++) in the prophylactic group (Figure 4G, Figure 5B) while it was strongly positive (+++) in the control (Figure 4E, Figure 5B) and treatment (Figure 4H, Figure 5B) groups and it was weak (+) in inflammation (Figure 4F, Figure 5B) group. The increase in caspase 3 immunoreactivity in the treatment group compared to the inflammation group was statistically significant (*p*=0.0436) (Figure 5B). These results suggested that using of favipiravir applied before the inflammation model may control the inflammation in intestinal enterocyte cells. In addition, the caspase 3-dependent apoptotic pathway may not be triggered or responsible for enterocytes in inflammation model cells.

**Figure 6:**
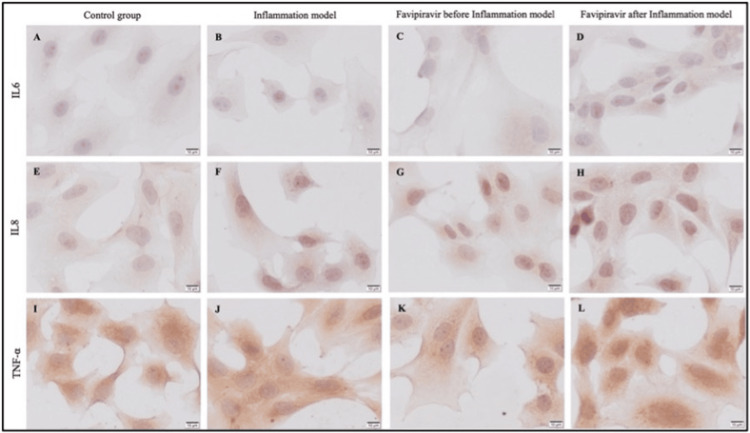
Assessment of IL-6, IL-8, and TNF-α through immunocytochemistry in the study groups. Immunoreactivity of IL6 (A-D), IL8 (E-H), and TNF-α (I-L) in control (A, E, I), inflammation model (B, F, J), before (prophylactic) (C, G, K) or after favipiravir applied (treatment) (D, H, L) inflammation model groups. Scale bars: 10 µm.

**Figure 7:**
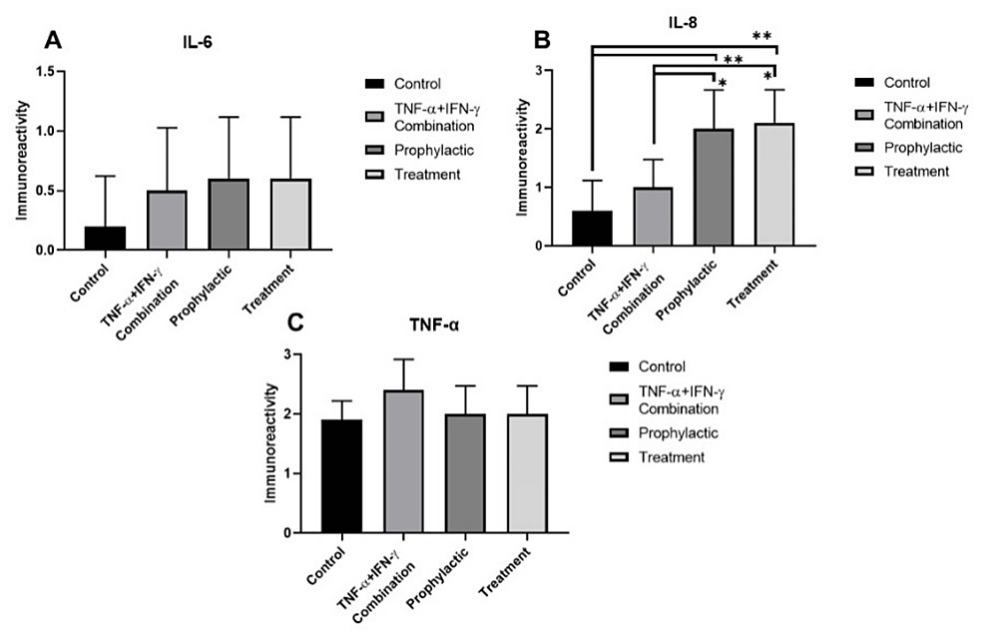
Statistical analysis of immunocytochemistry findings for IL-6, IL-8, and TNF-α within the study groups. IL6 (A), IL8 (B), and TNF-α (C) immunoreactivity measurements and statistical analysis of them in all experimental groups. The results are presented as mean ± SD and significance of difference indicated with asterisk (*) as *p* value smaller than 0.05 (*p*<0.05). Two asterisks (**) indicate *p* value smaller than 0.01 (*p*<0.01).

IL6 immunoreactivity was negative (-) in control (Figure 6A, Figure 7A). Weak (+) IL6 immunoreactivity was observed in inflammation, prophylactic, and treatment groups (Figure 6B-6D, Figure 7A). While IL8 immunoreactivity was negative (-) in control enterocytes (Figure 6E, Figure 7B), weak (+) IL8 immunoreactivity was detected in the inflammation model (Figure 6F, Figure 7B). In prophylactic (Figure 6G, Figure 7B), and treatment group enterocytes (Figure 6H, Figure 7B) moderate (++) immunoreactivity was detected. When the increase of IL8 immunoreactivity in prophylactic and treatment groups compared to the control group it was statistically significant (*p*=0,0060 and *p*=0,0044, respectively). Also, the prophylactic and treatment groups' IL8 immunoreactivity compared to the inflammation model group was statistically significant as well (*p*=0,0436 and *p*=0,0335, respectively). Strong (+++) TNF-α immunoreactivity was observed in inflammation model group enterocytes (Figure 6J, Figure 7C), it was moderate (++) and decreased in control, prophylactic, and treatment group cells (Figure 6I, Figure 6K, Figure 6L respectively, Figure 7C). 

**Figure 8:**
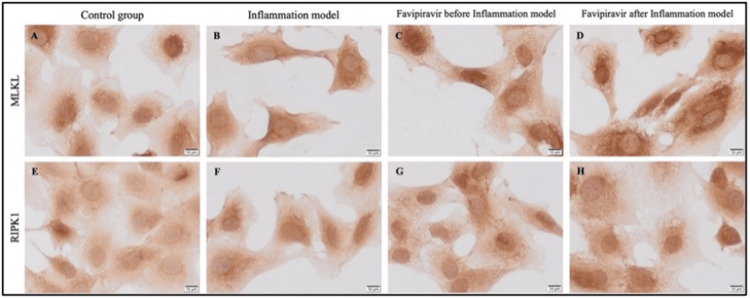
Evaluation of MLKL and RIPK1 via immunocytochemistry within the study groups. Immunoreactivity of MLKL (A-D) and RIPK1 (E-H) in control (A, E), inflammation model (B, F), before (prophylactic) (C, G) or after favipiravir applied (treatment) (D, H) inflammation model groups. Scale bars: 10 µm.

**Figure 9:**
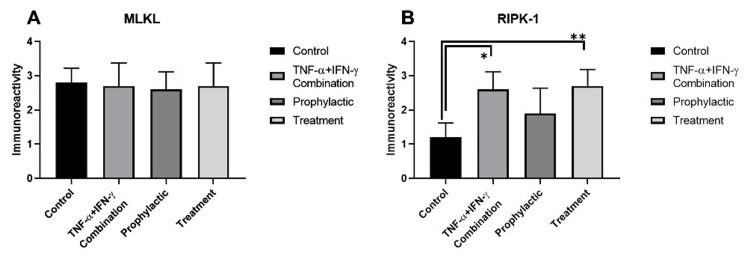
Statistical evaluation of immunocytochemistry results for MLKL and RIPK1 within the study groups. MLKL (A) and RIPK1 (B) immunoreactivity measurements and statistical analysis of them in all experimental groups. The results are presented as mean ± SD and significance of difference indicated with asterisk (*) as *p* value smaller than 0.05 (*p*<0.05). Two asterisks (**) indicate *p* value smaller than 0.01 (*p*<0.01).

MLKL immunoreactivity was observed in all groups (Figure 8A-8D, Figure 9A). However, strong (+++) immunoreactivity of MLKL was detected in all groups (Figure 8A-8D, Figure 9A). RIPK1 immunoreactivity was weak (+) in control enterocytes (Figure 8E, Figure 9B). While moderate (++) immunoreactivity of RIPK1 was observed in prophylactic group enterocytes (Figure 8G, Figure 9B), similar and strong (+++) immunoreactivity of RIPK1 were detected in the inflammation model (Figure 8F, Figure 9B) and treatment group enterocytes (Figure 8H, Figure 9B). The increase in RIPK1 immunoreactivity in the inflammation model group compared to the control was statistically significant (*1*=0,0194) (Figure 9B). Also, RIPK1 immunoreactivity was triggered in the treatment group compared to the control group and it was also statistically significant (*p*=0,0060) (Figure 9B).

## Discussion

Over the past years, the treatment of IBD has become more targeted, anticipating the use of immune-modifying therapies at an earlier stage [8]. This top-down approach has been correlated with favorable short and long-term outcomes, but it has also brought with it concerns regarding potential infectious complications [8]. Viral infections have emerged as a focal safety concern in patients with IBD, representing a challenge for the clinician [8].

Favipiravir, a purine nucleic acid analog and a virus RNA-dependent RNA polymerase inhibitor, is an antiviral medication that was approved in 2014 [21]. Favipiravir has played an important role in the treatment of influenza and Ebola in recent years [21,22]. In Japan, favipiravir has been used as a drug against influenza pandemics and as an option against SARS-CoV-2 under control [23]. However, the efficacy of antiviral treatments in the course of these patients especially after the trigger of inflammation has not been clearly elucidated [23]. 

The role of the gut microbiota in influencing lung diseases has been well described [24]. It is also known that respiratory virus infection causes perturbations in the gut microbiota [24]. They can explain gastrointestinal symptoms such as vomiting, abdominal pain, and diarrhea after influenza infection [7]. While the favipiravir drug is being applied for the treatment of patients with influenza, Ebola, and COVID-19, uncertainty remains about its effectiveness in treating GI system cells. In addition, after inflammation, the gut microbiota and enterocytes are also affected and the epithelial cells are damaged with cell death pathways. 

In our study, we investigated favipiravir treatment effects on inflammation mimicking models created by enterocytes with TNF-α and IFN-γ which causes cytokine storm symptoms [19,20]. Our results demonstrated that caspase 1 immunoreactivity was increased after favipiravir application compared to the control group while it did not change in the case of caspase 3. This may suggest that favipiravir treatment before the inflammation model may control the inflammatory caspase pathway in intestinal enterocyte cells and protect the enterocytes from the inflammatory response. Moreover, the caspase 3-dependent apoptotic pathway may not be triggered or responsible for enterocytes in the inflammation model.

One of the two inflammatory cytokines, IL6 and IL8, was also evaluated in this study. IL6 immunoreactivity was negative in control and showed weak immunoreactivity in inflammation model group, prophylactic and treatment group enterocytes. Therefore, it is thought favipiravir may trigger IL6 cytokine release to start to inflammatory pathway to trigger the other immune cells. 

IL8 immunoreactivity was also detected as negative in the control group and less in the inflammation model group compared to the prophylactic and treatment groups which may suggest that favipiravir may cause the increase of IL8 to cooperate with other interleukins or cytokines. 

MLKL and RIPK1 are responsible for the necroptosis [17]. According to our results, MLKL immunoreactivity was similar in all groups while RIPK1 immunoreactivity increased in the treatment group compared to the control group. Necroptosis is an inflammatory type of programmed cell death mediated by RIPK1/RIPK3/MLKL [17]. According to our results, in the treatment group enterocytes necroptosis was triggered through increasing MLKL, which is the main effector protein in necroptosis, and RIPK1 which is an important mediator of inflammation and cell death. 

Strong TNF-α immunoreactivity was observed in the inflammation model group enterocytes while it was detected as moderate in all other groups. Tanaka et al. showed that favipiravir suppresses TNF-α production in response to influenza virus infection which plays an important role in the pathogenesis of infection and has been correlated with the severity of infection [22]. Therefore, decreased immunoreactivity of TNF-α in inflammation-mimicking model cells was supporting cell death after favipiravir application. 

Yamamura et al. indicated that favipiravir could partially control inflammatory mediators but could not completely control them or their respiratory status [25]. This supports our inflammation and cytokine storm continuation after favipiravir administration. Also, according to our result, when the favipiravir was applied before the inflammation model it protected the cells from necroptosis. Moreover, through the caspase 1 mechanism, it controls the inflammation in the prophylactic group. Therefore, favipiravir application may be effective on enterocytes during treatment or protect them before inflammation storms.

Limitations

One potential limitation of this study is the absence of a cell proliferation assay for the four groups. Incorporating a cell proliferation assay could have provided conclusive evidence regarding the protective effect of favipiravir, whether it was observed during a cytokine storm or afterward.

## Conclusions

In conclusion, favipiravir was first time evaluated with inflamed enterocytes that may have some benefits, and these findings helped to inform a treatment strategy for severe gut inflammation and rescue of enterocytes from the cell death pathway. Long-term in vitro and in vivo studies could be needed to reveal the place of favipiravir in the treatment of enterocytes affected by inflammation.
